# Comparison of maternal and child health service performances following a leadership, management, and governance intervention in Ethiopia: a propensity score matched analysis

**DOI:** 10.1186/s12913-021-06873-8

**Published:** 2021-08-23

**Authors:** Mesele Damte Argaw, Binyam Fekadu Desta, Sualiha Abdlkader Muktar, Wondwosen Shiferaw Abera, Ismael Ali Beshir, Israel Ataro Otoro, Asegid Samuel, Deirdre Rogers, Kristin Eifler

**Affiliations:** 1 USAID Transform: Primary Health Care, JSI Research & Training Institute, Inc, Addis Ababa, Ethiopia; 2 Federal Ministry of Health, Health Extension and Primary Health Services Directorate, Addis Ababa, Ethiopia; 3 Federal Ministry of Health, Human Resource Development Directorate, Addis Ababa, Ethiopia; 4 JSI Research & Training Institute, Inc, Boston, USA

**Keywords:** Leadership, management, and governance, Performance improvement, Training, Ethiopia

## Abstract

**Background:**

Leadership, management, and governance (LMG) interventions play a significant role in improving management systems, enhancing the work climate, and creating responsive health systems. Hence, the Ethiopian Ministry of Health with the support of the USAID Transform: Primary Health Care project has been implementing LMG interventions to improve performances of primary healthcare entities. The purpose of this evaluation was to compare maternal and child health service performances and overall health system strengthening measurement results of primary health care entities by LMG intervention exposed groups.

**Methods:**

The study used a cross-sectional study design with a propensity matched score analysis, and was conducted from August 28, 2017, to September 30, 2018, in Amhara, Oromia, Tigray, and Southern Nations, Nationalities, and Peoples’ (SNNP) regions. Data collection took place through interviewer and self-administered questionnaires among 227 LMG intervention exposed and 227 non-exposed health workers. Propensity score matched analysis was used to balance comparison groups with respect to measured covariates.

**Results:**

The mean overall maternal and child health key performance indicator score with standard deviation (± SD) for the LMG intervention exposed group was 63.86 ± 13.16 and 57.02 ± 13.71 for the non-exposed group. The overall health system strengthening score for the LMG intervention exposed group (mean rank = 269.31) and non-exposed group (mean rank = 158.69) had statistically significant differences (U = 10.145, z = − 11.175, *p* = 0.001). In comparison with its counterpart, the LMG exposed group had higher average performances in 3.54, 3.51, 2.64, 3.00, 1.07, and 3.34 percentage-points for contraceptive acceptance rate, antenatal care, skilled birth attendance, postnatal care, full immunization, and growth monitoring services, respectively.

**Conclusion:**

There were evidences on the positive effects of the LMG intervention on increased maternal and child health services performances at primary healthcare entities. Moreover, health facilities with LMG intervention exposed health workers had higher and statistically significant differences in management systems, work climates, and readiness to face new challenges. Therefore, this study generated evidence for integrating LMG interventions to improve the performance of primary healthcare entities and maternal and child service uptake of community members, which contributes to the reduction of maternal and child deaths.

**Supplementary Information:**

The online version contains supplementary material available at 10.1186/s12913-021-06873-8.

## Introduction

Within the last couple of decades, evidence shows global improved health outcomes. However, this remarkable success significantly varies from place to place. In particular, the health systems of sub-Sahara Africa regions still show low levels of access to essential quality health services at an affordable cost [1–3]. Despite the reported substantial reductions in maternal and neonatal mortality rates, the region is still registering the highest rates throughout the world. The causes for this are complex and multifaceted and can be explained with determinants related to communities, health workforces, as well as the health and economic systems of countries. The World Health Organization (WHO) recommends applying an interlinked six building block approach to strengthen health systems [1]. Capacitating the leadership, management, and governance competencies of health workforces at all levels is believed to improve the organizational culture and achievements of proven public health interventions [1–3].

The Ministry of Health (MoH) recommends four pillars of excellence in health, namely: service delivery, quality improvement and assurance, leadership and governance, and health system capacity to achieve its vision of ‘healthy, productive, and prosperous Ethiopians’ [4]. Leadership, management, and governance (LMG) interventions thus play a significant role in improving management systems, enhancing the work climate, and creating responsive systems that are more accessible, with better quality and expanded service availability at affordable costs [5, 6]. Therefore, the Ministry and development partners apply a systematic approach to enhance the technical competence of healthcare providers and strive to increase the quality of services sustainably, and have them managed by a well-functioning primary healthcare system [7].

Ethiopia’s health system - as in many other low-income countries - lacks adequate infrastructure and resources. Health managers often lack the leadership, management, and governance (LMG) competencies and skills to overcome the numerous challenges associated with delivering proven, cost-effective health services, such as family planning, quality of care along the childbirth continuum, and childcare [2, 3, 8]. The country’s maternal and newborn health still needs improvement, with significant disparities in access to services and health outcomes. Although the proportion of mothers dying per 100,000 live births declined from 1400 in 1990 to 420 in 2013, the ratio is still unacceptably high [9]. Similarly, the infant mortality rate has declined from 59.6 per 1000 live births in 2009 to 36.5 per 1000 live births in 2019 [10].

Some studies have shown that the bottlenecks in the performance of health systems can be solved by building competencies of health workers related to leading, managing, and governing practices [3, 6, 11–15]. Therefore, Ethiopia has adopted and implemented LMG interventions to strengthen its health system and build services that are accessible to more people by developing inspired and inspiring leaders, establishing sound management systems, and ensuring transparent practices within and among individuals, networks, organizations, and governmental bodies.

However - to the knowledge of the investigators of this study - there are no studies which determine effects of the LMG intervention on maternal and child health service performance, using a propensity score matching (PSM) analysis. The investigators chose PSM for its merits in estimating the probability of receiving the intervention based on computed covariates. The method also ensures the reduction of selection bias and balance of comparison groups [16–18]. Therefore, the aim of this study was to determine the effects of leadership, management, and governance interventions on maternal and child health service performance at primary healthcare entities after implementing the intervention over the course of a one-year period.

## Materials and methods

### Study design

This study employed a cross-sectional study design with a propensity matched score analysis that measured the effects of the LMG intervention on maternal and child health service performance by LMG exposure statuses [19–21]. The PSM approach was used to explain possible differences at baseline variables between LMG exposed and non-exposed groups. The PSM aimed to balance the LMG intervention exposed and non- exposed groups with respect to measured baseline covariates, to achieve a comparison with reduced selection bias [16–18]. Hence, any observed differences on maternal and child health service coverages can demonstrate the effects of the LMG intervention. The study was conducted between August 28, 2017 and September 30, 2018, in Amhara, Oromia, SNNP, and Tigray regions of Ethiopia.

### Study setting

The Ethiopian health system is comprised of primary, secondary, and tertiary level institutions. The primary level of the health tier system encompasses district hospitals which cover 60,000–100,000 people and four to five health centers with an average targeted population of 25,000 people. In addition, each health center oversees an average of five satellite health posts with a target population of 3500 to 5000 people. The primary healthcare entities are mandated to provide preventive, curative, promotive, and rehabilitative health services to about 100,000 people. The secondary and tertiary levels manage complex heath conditions for larger size populations and include general and referral hospitals [4].

USAID Transform: Primary Health Care works closely with regional state health bureaus and aims to contribute to the successful achievement of the national goal of preventing child and maternal deaths (PCMD). To realize this aim, the project implements LMG related interventions that create conducive work environments, strengthen management systems, and create responsive healthcare providers and improve their capacity towards resource mobilization, allocation, and utilization [7]. within 4 years, (2017–2019) - through the direct support of the project - 184 districts and 519 health centers had LMG trained healthcare workers [7]. The trainees went on to develop 656 performance improvement projects and implemented them in their respective primary healthcare entities [22].

#### Description of the LMG intervention

The LMG intervention is dedicated to capacitating health workers with leading, managing, and governing practices. The USAID Transform: Primary Health Care project, in collaboration with regional health bureaus, randomly selected primary healthcare entities for enrollment into the intervention. Based on the course syllabus, trainees were informed about priority health areas, resource management and health service delivery management, and the concept of leadership, management, and governance for a result model of performance improvement [23]. The LMG interventions included didactic sessions lasting 6 days and implementation of performance improvement projects, supplemented with three to four coaching sessions and participation in knowledge sharing events.

During the six-day classroom training sessions, each primary healthcare entity, represented by three to four health workers, committed to work together for about 9 months. Each LMG team then developed maternal and child health service-related performance improvement projects. While developing the projects, teams used the ‘challenge model’ tool [5]. This tool helped the teams to identify their challenges through reviewing their organizational mission and strategic priorities, develop a shared vision, define a measurable result, assess their current situation to take accurate baseline measurements, identify obstacles and root causes (using a fishbone analysis, the why technique, or workflow analysis), define challenges in light of the root causes and select priority actions, develop an action plan that estimates the human, material, and financial resources needed as well as timelines for implementing the actions, and implement the plan and monitor and evaluate progress towards achieving the desired results [5, 23]. Some of the performance improvement projects developed and implemented by the trainees aimed to increase the proportion of institutional deliveries, the proportion of modern contraceptive users, and the proportion of well-baby service utilization (Additional file 1).

Upon returning back to their workplaces, the LMG core teams were instructed to organize a consensus building meeting on their performance improvement project, with participation from all health workers. Hence, staff of primary healthcare entities with shared visions worked together to achieve optimum results. During the implementation of each performance improvement project, each team received three to four onsite coaching sessions from experts [23] (Additional file 2). The coaches applied the observe, ask, listen, feedback and agree (OALFA) technique. At the end of the performance improvement projects, LMG intervention exposed health workers were invited to present their findings and lessons in knowledge sharing events organized at district level.

### Target population

Two groups were targeted, namely: LMG intervention exposed and non-exposed health workers employed in primary healthcare entities providing maternal and child health services within the four regions of Ethiopia.

### Eligibility criteria

Health workers who volunteered to participate and that were working within the USAID Transform: Primary Health Care projects’ targeted primary healthcare entities were enrolled in this study.

### Sampling size and sampling

The national average performance scores against selected maternal and child health indicators of primary healthcare entities were found to be at 65% [24]. With the assumption of training directives, LMG trained and capacitated healthcare workers improved the score of primary healthcare entities to 80% or more on the same key performance indicators (KPIs), and believing the presence of heterogeneity across regions and between primary healthcare entities, design effect of 2 was considered [25, 26]. The required sample size with a power of 90% to detect the effects of the LMG intervention for exposed (n_1_ = 272) and non-exposed (n_2_ = 272) groups was 544. The researchers used a database as a sampling frame to select primary healthcare facilities using systematic random sampling methods. Following this step, data collectors randomly chose one of three key persons using a lottery method, namely: head of health centers, maternal and child health core process owners, and health center - health post linkage focal persons.

### Data collection

The questionnaires were prepared after a thorough review of relevant literature and the national leadership, management, and governance in-service training materials for hospitals and health center managers [3, 23, 27]. The questionnaires were developed in English (Additional file 3) and translated into local languages (i.e., Amharic, Afan Oromo, and Tigrigna), then translated back into English. Data were collected from LMG intervention exposed and non-exposed healthcare providers using interview and self-administered structured questionnaires. Sixteen data collectors, each with clinical, health management, social science, or public health training were recruited. Data collectors and supervisors were trained on ethical principles, data collection tools, and interviewing techniques. Before the actual data collection began, all tools were piloted and amended accordingly.

#### Outcome measurements

Six maternal and child health key performance indicators endorsed by the Ministry of Health [24], and three categories of immediate outcomes on the leading, managing, and governing model for results measurements were considered in the study [5, 23]. The detailed characteristics of selected KPIs are discussed after which the features of the health system strengthening variables are presented.

#### Maternal and child health performance

Data were extracted from health facility routine health information management systems. Six maternal and child health program performance KPIs were selected as dependent variables. These indicators were: (1) contraceptive acceptance rate (CAR), (2) antenatal care (ANC), (3) skilled (institutional) birth attendance (SBA), (4) postnatal care (PNC), (5) full immunization coverage for infants under the age of 1, and (6) growth monitoring for children 2–59 months old. Each indicator was scored from 0 to 100 percentage points. The average of all six indicators was considered as an overall maternal and child health performance score.

#### Health system strengthening survey

Eighteen questions, with a 10-point Likert scale [28] were used to measure responses to statements presented on: strengthened management systems (9 items), enhanced work climate (5 items), and responsiveness or capacity of the health system to overcome new challenges (4 items). The sample questionnaire for each category is presented in Table 1. The respondents were LMG intervention exposed and non-exposed health workers operating within primary healthcare entities.

**Table 1:** Sample questionnaires

Serial no.	Sample tool	Category
WC1	In this office, employees understand the organizational structure and reporting lines of their unit/department and how their job functions relate to overall departmental objectives and goals.	Strengthened management systems.
WC10	My contributions at work are acknowledged and appreciated.	Enhanced work climate.
WC13	In this office, supervisors delegate challenging assignments to assistants, which helps them to develop their skills and expertise.	Responsiveness or capacity of health system to overcome new challenges.

The box presents sample items from composite scales, representing the three categories namely: management systems, work climates, and responsiveness

#### Independent variables

Data on possible independent variables were collected. Individual characteristics of the study participants which included age, gender, profession, educational level, service years, and salary were captured. In addition, primary healthcare entity characteristics such as access to roads, distance from zone capital in kilometers, and catchment population were collected.

### Data analysis

The data were analyzed using the Statistical Package for Social Sciences (SPSS) version 25 [29]. A PSM analysis was conducted using the R-plugin for propensity score matching for SPSS [30] [31]. The internal reliability of the tools was assessed using Cronbach’s alpha values [32]. According to Bland et al. [33], if the Cronbach’s alpha value score is more than 0.7, the scale can be considered reliable. The tools have 18 questions divided into three categories, namely: strengthened management systems (9 questions), enhanced work climate (5 questions), and the capacity to respond to challenges (4 questions). The reliability test results were 0.839 for work climate, 0.895 for strengthened management systems, and 0.886 for the capacity to respond to changes, which showed the scale used was internally consistent and reliable.

A statistical test using multi-collinearity analysis through determining the variance inflation factor (VIF) was run to check the tools’ divergent validity [20]. According to Menard [34], if the VIF reported value exceeds 10, it implies the associated regression coefficients are poorly estimated because of multi-collinearity. In this study, the collinearity test VIF results were: 1.018 for work climate, 2.94 for strengthened management systems, and 6.443 for capacity to respond to new challenges and no VIF value exceeded 10. Hence, there was no observed multi-collinearity affecting the regression coefficients.

PSM is a single score used to balance the LMG intervention exposed and non-exposed groups on observed covariates [30]. In this study, the covariates included in the PSM model are the socio-demographic characteristics of health workers including gender, age, profession, salary, marital status, and tenured service years. In addition, the researcher included the characteristics of the targeted primary healthcare entities, regions, catchment populations, distance from zone capitals in kilometers, and access to all weather roads in the PSM model. During matching, a standardized difference of covariates between the two groups of less than 10% was taken as adequate. Using a logistics regression estimation (logit model), the PSM was analyzed by assigning 1 for LMG intervention exposed and 0 for non-exposed groups, with nearest neighbor matching, caliper of 0.2 and matching ratio of 1 to 1. The relative multivariate imbalance L1 was 0.926 before matching and 0.916 after matching [35]. Only ‘place of work’ had a large imbalance after PSM with |d| = 0.80, which violates the recommended criteria standard error difference of |d| < 0.25 (Additional file 4).

Finally, using PSM, data from 454 (227 LMG intervention exposed and 227 non-exposed) participant groups were used to analyze and compare maternal and child health service performances of primary healthcare facilities (Additional file 5). Descriptive statistics were employed using frequencies and tables. The homogeneity of the data was checked using Levene’s test for equality of variances. The results revealed that maternal and child health coverage data were found to be homogeneous at *p* > 0.05. While the data on strengthening management system, enhanced work climate, and capacity to respond to new challenges were found to be non-homogeneous at *p* < 0.05 (Additional file 6).

An independent sample t-test was analyzed to check statistical difference on maternal and child health service performance coverage between LMG intervention exposed and non-exposed groups. In addition, a non-parametric test, (Mann-Whitney U test) was conducted to check presence of significant differences in mean rank LMG intervention exposed and non-exposed groups [31].

### Ethical considerations

Ethical clearance was obtained from four regional institutional review boards. Ethical clearances were granted by the Amhara Public Health Institute, (Ref. No. HRTT02/137/2018), the Oromia Regional State Health Bureau, (Ref. No. BEFO/HBTPH/1-8/476), the SNNP Regional State Health Bureau, (Ref, No. PLMG-19/8407), and the Tigray Regional State Health Bureau’s (Ref. No. 453/1418/10) institution review boards and research ethics committees. Data were collected after getting full informed written consent from each participant and facility manager. Privacy, anonymity, and confidentiality were maintained throughout the data collection, analysis, and report writing activities. This study has no known risk and no payment was made to participants.

## Results

Table 2 presents the background socio-demographic characteristics of the study participants categorized by LMG exposure, before and after propensity score matched analyses. A total of 544 primary healthcare entities that have 272 LMG intervention exposed and 272 non-exposed groups were enrolled in this study. However, after PSM, only data from 454 primary healthcare entities were included in the final analysis. The data analysis revealed that before PSM, there were significant differences on distribution of age categories (*p* < 0.002), places of work (*p* < 0.001) and years of service (*p* < 0.001) tenured by the study participants. After PSM analysis, only the distribution of ‘place of work’ was found to be statistically different at *p* < 0.001.

**Table 2:** Socio-demographic characteristics of study participants, by LMG exposure status and PSM, September 2018

Variable	Category	Before propensity score matching LMG interventions					Total (***N*** = 544)		After propensity score matching LMG interventions					Total (***N*** = 454)	
		Exposed (*N* = 272)		Non exposed (*N* = 272)		*p- value*			Exposed (*N* = 227)		Non-exposed (*N* = 227)		*p- value*		
		N	%	N	%		N	%	N	%	N	%		N	%
Region	Amhara	80	29%	71	26%	0.736	151	28%	59	26%	59	26%	0.324	118	26%
	Oromia	75	28%	77	28%		152	28%	68	30%	67	30%		135	30%
	SNNP	83	31%	93	34%		176	32%	69	30%	81	36%		150	33%
	Tigray	34	13%	31	11%		65	12%	31	14%	20	9%		50	11%
Sex	Male	201	74%	202	74%	0.922	403	74%	168	74%	171	75%	0.922	339	75%
	Female	71	26%	70	26%		141	26%	59	26%	56	25%		115	25%
Age	< 30 years	171	63%	209	77%	0.002	380	70%	163	72%	176	78%	0.367	339	75%
	31–40 years	74	27%	46	17%		120	22%	56	25%	44	19%		100	22%
	41–50 years	16	6%	14	5%		30	6%	8	4%	7	3%		15	3%
	51+ years	11	4%	3	1%		14	3%							
Profession	BSc nurse	34	13%	38	14%	0.767	72	13%	28	12%	26	11%	0.997	54	12%
	BSc public health officer	68	25%	55	20%		123	23%	46	20%	45	20%		91	20%
	Diploma nurse	121	44%	122	45%		243	45%	109	48%	108	48%		217	48%
	Midwife (BSc and diploma)	23	8%	30	11%		53	10%	23	10%	25	11%		48	11%
	MSc health services management	12	4%	13	5%		25	5%	9	4%	9	4%		18	4%
	Other	14	5%	14	5%		28	5%	12	5%	14	6%		26	6%
Salary	< 3500 ETB^*^	77	28%	91	33%	0.331	168	31%	72	32%	76	33%	0.662	148	33%
	3501–8540 ETB	164	60%	157	58%		321	59%	135	59%	136	60%		271	60%
	8541+ ETB	31	11%	24	9%		55	10%	20	9%	15	7%		35	8%
Marital status	Single	89	33%	95	35%	0.425	184	34%	78	34%	83	37%		161	35%
	Married	181	67%	172	63%		353	65%	147	65%	141	62%		288	63%
	Separated	2	1%	5	2%		7	1%	2	1%	3	1%		5	1%
Years of service	< 5 years	134	49%	158	58%	0.001	292	54%	118	52%	131	58%	0.220	249	55%
	6–21 years	138	51%	104	38%		142	26%	109	48%	96	42%		205	45%
	22+ years	0	0%	10	4%		10	2%							

Table 2 depicts socio-demographic characteristics of the study participants before and after propensity score matched analysis

### Comparison of maternal and child health performances

An independent sample t-Test was applied with seven variables after confirming the assumption of homogeneousness of the data set using Levene’s test (*p* > 0.05). The mean overall maternal and child health KPIs score [± standard deviation (SD)] was 63.86 ± 13.16 and 57.02 ± 13.71 among LMG intervention exposed and non-exposed groups, respectively. The performance score was significantly higher among the LMG intervention exposed group than the non-exposed group with t = 5.43, df = 452, *p* < 0.001. The difference of 6.84 percentage-points in the average maternal and child health KPIs had statistically significant differences between the LMG intervention exposed and non-exposed groups (Table 3).

**Table 3:** Maternal and child health service KPI scores by LMG exposure status using an independent samples test, 2018

	t-test for Equality of Means										
	LMG exposed *N* = 227		Non-LMG exposed *N* = 227		T	df	Sig. (2-tailed)	Mean difference	Std. error difference	95% confidence interval of the difference	
	Mean	SD	Mean	SD						Lower	Upper
Contraceptive acceptance rate (CAR)	82.47	16.59	75.96	16.38	4.21	452	0.001	6.50	1.55	3.47	9.55
Antenatal care (ANC)	69.75	26,37	60.65	24.64	3.79	452	0.001	9.09	2.39	4.39	13.81
Skilled birth attendance (SBA)	56.28	22.97	46.39	22.26	4.66	452	0.020	9.89	2.12	5.72	14.06
Postnatal care (PNC)	64.42	22.2	56.12	21.92	4.01	452	0.011	8.30	2.07	4.23	12.37
Full immunization (FI)	80.41	15.12	78.00	15.31	1.69	452	0.093	2.40	1.43	−0.40	5.22
Growth monitoring (GM)	37.83	23.42	24.98	23.48	3.56	452	0.678	7.84	2.20	3.52	12.17
Overall KPI score	63.86	13.16	57.02	13.71	5.43	452	0.001	6.84	1.26	4.36	9.32

Table 3 presents the maternal and child health service key performance indicators scored by LMG exposure statuses. In addition, the relationship between LMG exposure and performance were statistically determined using an independent sample t-test

Four variables were found non-homogeneous on Levene’s test (*p* < 0.05) and a non-parametric independent samples Mann–Whitney U test was applied to check for differences in performance of primary healthcare entity scores between LMG intervention exposed and non-exposed groups. The results revealed that the overall work climate scores for LMG intervention exposed (mean rank = 296.31) and non-exposed (mean rank = 158.69) were statistically different, U = 10,145, Z = − 11.175, *p* = 0.001 (Table 4).

**Table 4:** Health system strengthening mean rank test using Mann-Whitney U Test, 2018

	Strengthened management system (A)		Enhanced work climate (B)		Capacity to respond to new challenges (C)		Overall work climate score (A) + (B) + (C)	
	LMG exposed	Non-exposed	LMG exposed	Non-exposed	LMG exposed	Non-exposed	LMG exposed	Non-exposed
N	227	227	227	227	227	227	227	227
Mean rank	312.71	142.29	259.52	195.48	263.99	191.01	296.31	158.69
Sum of ranks	70,984.50	32,300.50	58,911.50	44,373.50	59,925.50	43,359.50	67,262.00	36,023.00
Mann-Whitney U	6422.500		18,495.500		17,481.500		10,145.000	
Wilcoxon W	32,300.500		44,373.500		43,359.500		36,023.000	
Z	−13.840		−5.220		−5.929		−11.175	
Asymp. sig. (2-tailed)	.001		.001		.001		.001	

Table 4 presents a non-parametric statistical test which depicts the relationship between LMG exposure and health system strengthening scores

## Discussion

This comparative cross-sectional study using PSM analysis was conducted to evaluate the USAID Transform: Primary Health Care project supported LMG intervention in Ethiopia. The results revealed that LMG intervention exposed primary health care facilities had a 6.84% higher maternal and child health service performance coverage with statistically significant differences form their counterpart. The results of this study generate evidences for policy makers, development partners, and health workers to strategize and adapt proven interventions to improve access to and quality of maternal and child health services, enhance work climates, and prepare health workers to face new challenges at primary healthcare facilities [5, 23].

In this study, we found a significant positive gain in maternal and child health service KPI scores. More specifically, the LMG intervention had a positive association with contraceptive acceptance rates, skilled birth attendance, postnatal care, and growth monitoring service coverages. Hence, the multi-faceted LMG intervention addresses some gaps in desired competencies and skills of health workers which enables them to solve existing challenges in their workplace. These findings were consistent with other similar studies documented that show significant increases of health service coverage as a result of LMG training and interventions in Zambia, Gambia, Egypt, Ethiopia, Ghana, Kenya and Mozambique [3, 6, 11–15, 36, 37].

Overall, the LMG intervention increased maternal and child health KPIs by 4.35%. This gain might be a result of the capacity building interventions in leading, managing, and governing practices. This finding is consistent with the Desta et al. [37] comparative reports of LMG intervention districts which were better at capacitating health workers, strengthening teams, and availing inputs than non-LMG intervention districts. Similarly, La Rue et al. [11], confirm that health service coverages were better in intervention districts than non-intervention districts through strengthening of leadership and management skills.

Primary healthcare entities with LMG intervention exposed health workers had significantly better and higher scores in management systems, creating good work climates and preparedness with capacity to respond for new challenges than non-exposed facilities (*p* < 0.001). These findings correspond with the Mutale et al. [3] report on the catalyzing effects of leadership and management trainings on health system strengthening in Zambia. Similarly, the findings also concur with La Rue et al. [11], who assessed and compared baseline, end-line, and post-intervention data and found an enhanced work climate through leadership development programs in Kenya. Rowe et al. [38] also confer through a systematic review that professional healthcare provider practices improved using multiple strategies rather than by a single strategy. Similarly, Arinez et al. [39] confer that good leadership and management practices improve the work climate, staff satisfaction, motivation, and performances.

## Limitations of the study

Like many studies, this study has limitations. The socio-demographic characteristics of LMG exposed and non-exposed groups significantly differ before the PSM. Hence, 45 pairs of individual data were dropped for lacking similar covariate characteristics. In addition, the health service coverage reports were extracted from a routine health information system database, which has known limitations in completeness. Since, there were no baseline data, this study could not show the real effects using Difference–in–Difference (DID) matching techniques. Furthermore, the reported average higher scores on maternal and child health service indicators might be affected by other covariates which were not included in our PSM model.

## Conclusions

The LMG intervention had a positive and statistically significant impact on maternal and child health service coverages in comparison with non-LMG intervention primary healthcare entities. In addition, primary healthcare entities with LMG intervention exposed health workers had significantly higher scores in terms of management systems, work climate, and readiness to face new challenges.

Therefore, the LMG interventions were found to be effective in improving maternal and child health services through enhancing the LMG competencies and skills for health workers. It is recommended that LMG interventions be integrated at primary healthcare entities in order to accelerate the implementation of prioritized maternal and child health services and achieve the Sustainable Development Goals as a global target.

## Supplementary Information

**Additional file 1.** Sample performance improvement project against results documented in selected primary healthcare facilities, September 2018.

**Additional file 2 **Description of block and segmented LMG training course curriculum and interventions, September 2018**.**

**Additional file 3.** Data collected forms, September 2018.

**Additional file 4.** Propensity matched score analysis, September 2018.

**Additional file 5.** Data set, September 2018.

**Additional file 6.** Levene’s test of equality of variances, September 2018.

## Data Availability

All relevant data are within the paper and its Supporting Information files.

## Abbreviations

CBHI: Community-based health insurance
DMR: Desired measurable result
FMOH: Federal Ministry of Health
LMG: Leadership, management, and governance
OALFA: Observe, ask, listen, feedback and agree
SD: Standard deviation
SNNP: Southern Nations and Nationalities of Peoples’
SPSS: Statistical Package for Social Science
USAID: United States Agency for International Development
VIF: Variance inflation factor
WHO: World Health Organization
ZHD: Zonal health department
