# Supplementary material for: Comparison of maternal and child health service performances following a leadership, management, and governance intervention in Ethiopia: a propensity score matched analysis
Source: BMC Health Serv Res. 2021 Aug 23;21:862. doi: 10.1186/s12913-021-06873-8 (PMC8383359; doi:10.1186/s12913-021-06873-8)
Supplement: Supplementary file 1 — Additional file 1. Sample performance improvement project against results documented in selected primary healthcare facilities, September 2018. [file 12913_2021_6873_MOESM1_ESM.docx]

Additional file 1: Sample leadership projects against results documented in selected primary healthcare facilities, September 2018

| Region | Topic | Challenges/projects | Reported results (Aug 2017–Sept 2018) |
| --- | --- | --- | --- |
| Amhara | Woreda standards | June 2018: increase woreda management standards met from 53% to 75%. (HSS project, #21) | Increased woreda management standards met from 53% to 96%. |
|  | ANC4+ service uptake | June 2018: increase ANC4+ service uptake coverage from 48% to 75%. (Maternal and neonatal health: ANC project, #60) | Increased ANC4+ service uptake coverage from 48% to 58%. |
|  | Skilled delivery | June 2018: increase skilled delivery service uptake coverage from 64% to 70%. (Maternal and neonatal health: delivery project, #7) | Increased skilled delivery service uptake coverage from 64% to 71%. |
|  | Immunization | Aug 2018: decrease Penta3 defaulter rate from 7% to 5%. (Child health- immunization, #69) | Decreased Penta3 defaulter rate from 7% to 3%. |
|  | Severe acute malnutrition | Aug 2018: decrease severe acute malnutrition (prevalence rate from 3.6% to 1%. (Nutrition project, #75) | Decreased severe acute malnutrition prevalence rate from 3.6% to 1.3%. |
| Oromia | Disease Prevention | By end of Feb 2018, increase TB case detection rate from 40% to 60%. (Other: TB prevention and control project, #122) | TB case detection rate remained 40% (did not meet the desired measurable result). |
|  | Community-based health insurance | By end of Oct 2018, increase proportion of households enrolled in CBHI scheme from 8% to 75%. (CBHI project, #156) | Increased the proportion of households enrolled in CBHI scheme from 8% to 25%. |
|  | Disease prevention | By end of July 2018, decrease the prevalence malaria positivity rate from 2.8% to 0.8%. (Other: malaria prevention and control project, #147) | Decreased the prevalence malaria positivity from 2.8% to 0.7%. |
| SNNP | Family Planning | By end of December 2017, increase modern contraceptive acceptance rate from 34% to 60%. (FP project, #215) | Increased modern contraceptive acceptance rate from 34% to 67%. |
|  | Community-based health insurance | By end of June 2018, increase the proportion of households enrolled in CBHI scheme from 15% to 100%. (CBHI project, #241) | Increased the proportion of households enrolled in CBHI scheme from 15% to 64%. |
| Tigray | Hospital Reform | By end of Feb 2018, increase hospital services transformation guidelines standards met from 30% to 70%. (HSS project, #93) | Increased hospital services transformation guidelines standards met from 30% to 69%. |
