# Supplementary material for: Comparison of maternal and child health service performances following a leadership, management, and governance intervention in Ethiopia: a propensity score matched analysis
Source: BMC Health Serv Res. 2021 Aug 23;21:862. doi: 10.1186/s12913-021-06873-8 (PMC8383359; doi:10.1186/s12913-021-06873-8)
Supplement: Supplementary file 2 — Additional file 2 Description of block and segmented LMG training course curriculum and interventions, September 2018. [file 12913_2021_6873_MOESM2_ESM.docx]

Additional file 2: description of block and segmented LMG training course curriculum, and interventions, September 2018

Block course: a six-day classroom LMG training and six- to-nine-months-long leadership project organized for maternal health program champions from primary healthcare facilities. During the project period, the trainees received coaching from staff of in-service training institutes every 30–45 days.

Block approach course description

| Ser. no. | Day/description |
| --- | --- |
|  |  |
| 1 | Day 1 (7 hours) |
| 2 | Registration and welcome  Introductions, workshop expectations, ground rules pre-test **(120 minutes)** |
| 3 | Module 1: Overview and context of the health system in Ethiopia |
| 3.1. | UNIT 1: Existing and emerging healthcare issues and trends **(60 minutes)** |
| 3.2. | UNIT 2: Health systems strengthening **(60 minutes)** |
| 3.3. | UNIT 3: Health policy, strategies, and reforms in Ethiopia **(75 minutes)** |
| 4 | Module 2: Introduction to leadership, management, and governance |
| 4.1. | UNIT 1: Concepts and practices of leading, managing, and governing **(90 minutes)** |
| 4.2. | Closing and daily course evaluation **(30 minutes)** |
| 4.3. | Day 2 (6 hours) |
| 4.4. | Recap of day 1 sessions **(30 minutes)** |
| 4.5. | UNIT 1: Concepts and practices of leading, managing, and governing **(285 minutes)** |
| 4.6. | UNIT 2: Mobilizing communities and other stakeholders to improve health **(30 minutes)** |
| 4.7. | Closing and daily course evaluation **(15 minutes)** |
| 5. | Day 3 (6 hours) |
| 5.1. | Recap of day 2 sessions **(30 minutes)** |
| 6 | Module 3: Improving performance through enhanced leadership, management, and governance |
| 6.1. | UNIT 1: Participatory leading and managing practices **(195 minutes)** |
| 6.2. | UNIT 2: Moving from vision to action **(180 minutes)** |
| 6.3. | Closing and daily course evaluation **(15 minutes)** |
| 7. | Day 4 (6 hours) |
| 7.1. | Recap of day 3 sessions **(30 minutes)** |
|  | UNIT 3: Aligning, mobilizing and inspiring health workers for results **(375 minutes)** |
| 7.2 | Closing and daily course evaluation **(15 minutes)** |
| 8. | Day 5 (6 hours) |
| 8.1. | Recap of day 4 sessions **(30 minutes)** |
| 8.2. | UNIT 4: Improving participatory governance in health facilities **(195 minutes)** |
| 9 | Module 4: Resource management |
| 9.1 | UNIT 2: Management of health information **(90 minutes)** |
| 10 | Module 5: Health service delivery management |
| 10.1 | UNIT 1: Health service delivery management **(90 minutes)** |
| 10.2 | Closing and daily course evaluation **(15 minutes)** |
| 10.3 | Day 6 (4.5 hours) |
| 10.4 | Recap of day 5 sessions **(30 minutes)** |
| 10.5 | Finalization of LMG worksheets, present and submit **(180 minutes)** |
| 10.6 | Workshop evaluation, post-test and closing remarks **(60 minutes)** |
| 11. | Coaching |
| 11.1 | Session 1 |
| 11.2 | Session 2 |
| 11.3 | Session 3 |
| 11.4 | Session 4 |
| 12 | Knowledge sharing event |

*Segmented course: two workshops of three-day classroom LMG training, and six- to-nine months-long leadership project.*

Segment 1: two or three health system managers recruited from primary healthcare facilities (i.e., woreda health office, health centers, primary hospitals) attend two rounds of training with a one-month gap and implement 6-month-long leadership projects. The training is held at the district level, and every 30–45 days. Coaching is provided by zonal (regional) health departments (ZHDs).

Segment II: two or three health system managers from primary healthcare facilities (i.e., woreda health office, health centers, primary hospitals) attend two rounds of training with a 1–2-month gap and implement 6–9 month-long leadership projects. Every 30–45 days, the coaching is provided by USAID Transform: PHC Activity field staff.

Segmented approach course description

| Ser. no. | Day/description |
| --- | --- |
|  |  |
| 1 | Workshop I |
| 1 | Day 1 (7 hours) |
| 2 | Registration and welcome  Introductions, workshop expectations, ground rules pre-test **(120 minutes)** |
| 3 | Module 1: Overview and context of the health systems in Ethiopia |
| 3.1. | UNIT 1: Existing and emerging healthcare issues and trends **(60 minutes)** |
| 3.2. | UNIT 2: Health system strengthening **(60 minutes)** |
| 3.3. | UNIT 3: Health policy, strategies, and reforms in Ethiopia **(75 minutes)** |
| 4 | Module 2: Introduction to leadership, management, and governance |
| 4.1. | UNIT 1: Concepts and practices of leading, managing, and governing **(90 minutes)** |
| 4.2. | Closing and daily course evaluation **(30 minutes)** |
| 4.3. | Day 2 (6 hours) |
| 4.4. | Recap of day 1 sessions **(30 minutes)** |
| 4.5. | UNIT 1: Concepts and practices of leading, managing, and governing **(285 minutes)** |
| 4.6. | UNIT 2: Mobilizing communities and other stakeholders to improve health **(30 minutes)** |
| 4.7. | Closing and daily course evaluation **(15 minutes)** |
| 5. | Day 3 (6 hours) |
| 5.1. | Recap of day 2 sessions **(30 minutes)** |
| 6 | Module 3: Improving performance through enhanced leadership, management, and governance |
| 6.1. | UNIT 1: Participatory leading and managing practices **(195 minutes)** |
| 6.2. | UNIT 2: Moving from vision to action (**180 minutes)** |
| 6.3. | Closing and daily course evaluation **(15 minutes)** |
|  | Workshop 2 |
|  | Registration and welcome  Introductions, workshop expectations, ground rules pre-test **(120 minutes)** |
| 7. | Day 1 (6 hours) |
| 7.1. | Recap of day 3 sessions **(30 minutes)** |
|  | UNIT 3: Aligning, mobilizing and inspiring health workers for results **(375 minutes)** |
| 7.2 | Closing and daily course evaluation **(15 minutes)** |
| 8. | Day 2 (6 hours) |
| 8.1. | Recap of day 4 sessions **(30 minutes)** |
| 8.2. | UNIT 4: Improving participatory governance in health facilities **(195 minutes)** |
| 9 | Module 4: Resource management |
| 9.1 | UNIT 2: Management of health information **(90 minutes)** |
| 10 | Module 5: Health service delivery management |
| 10.1 | UNIT 1: Health service delivery management **(90 minutes)** |
| 10.2 | Closing and daily course evaluation **(15 minutes)** |
| 10.3 | Day 3 (4.5 hours) |
| 10.4 | Recap of day 5 sessions **(30 minutes)** |
| 10.5 | Finalization of LMG worksheets, present and submit **(180 minutes)** |
| 10.6 | Workshop evaluation, posttest and closing remarks **(60 minutes)** |
| 11. | Coaching |
| 11.1 | Session 1 |
| 11.2 | Session 2 |
| 11.3 | Session 3 |
| 11.4 | Session 4 |
| 12 | Knowledge sharing event |
