# Supplementary material for: Comparison of maternal and child health service performances following a leadership, management, and governance intervention in Ethiopia: a propensity score matched analysis
Source: BMC Health Serv Res. 2021 Aug 23;21:862. doi: 10.1186/s12913-021-06873-8 (PMC8383359; doi:10.1186/s12913-021-06873-8)
Supplement: Supplementary file 3 — Additional file 3. Data collected forms, September 2018. [file 12913_2021_6873_MOESM3_ESM.docx]

Additional file 3: Data collection tools.

**Survey introduction**

This study is aims to determine the effects of LMG training and intervention on maternal and child health service performance in four regional states of Ethiopia. USAID Transform: Primary Health Care will analyze the data and synthesize the information to select and modify the three training approaches, contents, time allocated, communication, and resources. Please note that the information you give us will be kept confidential and used only to modify the LMG intervention. The tool has three parts: part one is dedicated to ascertaining the socio-demographic characteristics of trainees; part two is dedicated to capturing information on independent and dependent variable ratings; and the third part is dedicated to collecting secondary data from training reports.

| **SN** | **Question** | **Coding categories** |
| --- | --- | --- |
| **I** | **Part I: Basic information** | |
| 1.1 | Date of assessment | __ __/__ __/__ __ __ __(DD/MM/YYYY) |
| 1.2 | Region | 1. Tigray 2. Amhara 3. Oromia 4. SNNP |
| 1.3 | Organization name |  |
| 1.4 | Catchment population | [ ] number |
| 1.5 | Distance in kilometers | [ ] Km |
| 1.6 | Access to roads | 1. Yes 2. No |
| 1.7 | Level of organization | 1. Primary hospital 2. Health center 3. Woreda health office 4. Zone health department 5. Other (specify ……………………………) |
| 1.8 | Age [in full years] | [ ] years |
| 1.9 | Sex | 1. Male 2. Female |
| 1.10 | Marital status | 1. Single 2. Married 3. Separated/divorced 4. Other (state)-------------------------- |
| 1.11 | Profession | 1. BSc nurse 2. BSc public health officer 3. Diploma nurse 4. MSC in health service management 5. Midwife nurse 6. Other (specify…………………………..) |
| 1.12 | Monthly income in ET Birr | [ ] Et Birr |
| 1.13 | Did you participate in LMG interventions? | 1. Yes 2. No |

**Health system strengthening measurements**

| III | Part III: Rating the health systems strengthening measurements. The LMG exposed and non-exposed health workers should rate based on their experience within their respective health facilities. The following questions measure the health system strengthening interventions. Please respond **by rating each statement that best describes the extent of your level of perception against 10 Likert scale.** The scale ranges from excellent (10) to poor (1).  Before and after   \| Excellent \| 10 \| 9 \| 8 \| 7 \| 6 \| 5 \| 4 \| 3 \| 2 \| 1 \| Poor \| \| --- \| --- \| --- \| --- \| --- \| --- \| --- \| --- \| --- \| --- \| --- \| --- \| | | | |
| --- | --- | --- | --- | --- | --- | --- | --- | --- | --- | --- | --- | --- | --- | --- | --- | --- |
| Category | Item code | Questions | Rate | Remark |
| Work Climate (WC) | WC1 | In this office employees understand the organizational structure and reporting lines of their unit/department, and how their job functions relate to overall departmental objectives and goals. |  |  |
|  | WC2 | For most meetings in this office, agendas are circulated to all before the meetings. |  |  |
|  | WC3 | For most meetings in this office, minutes are circulated to all soon after the meetings, indicating follow-up items. |  |  |
|  | WC4 | The leadership here keeps staff well informed about what is going on with the organization. |  |  |
|  | WC5 | In this office, cooperation and teamwork between staff in different units is encouraged. |  |  |
|  | WC6 | In this office, we are encouraged to use data to guide decision-making, priority-setting, and planning. |  |  |
|  | WC7 | In this office, we are encouraged to analyze problems carefully to understand root causes before deciding on solutions. |  |  |
|  | WC8 | In this office, formal individual performance appraisals are routinely conducted on an annual basis. |  |  |
|  | WC9 | In this office, supervisors provide constructive feedback to their assistants on a regular basis to help improve job performances. |  |  |
|  | WC10 | My contributions at work are acknowledged and appreciated. |  |  |
|  | WC11 | My supervisor works with me to identify my training needs and ensures I get the training or mentorship I need to do my job effectively. |  |  |
|  | WC12 | In this office, when staff attend trainings, effort is made to ensure that they apply what they have learned back at the job site |  |  |
|  | WC13 | In this office, supervisors delegate challenging assignments to assistants, which helps them to develop their skills and expertise. |  |  |
|  | WC14 | In this office, when giving special assignments, supervisors clearly communicate expectations at the beginning and check in on progress, without micromanaging. |  |  |
|  | WC15 | In this office, supervisors or unit leaders regularly monitor progress and hold every staff accountable for following through on assigned tasks related to work plans. |  |  |
|  | WC16 | In this office, supervisors do everything in their power to help resolve HR issues (such as confirmation) in a timely manner. |  |  |
|  | WC17 | In this office, supervisors take appropriate corrective action when an employee is not performing well. |  |  |
|  | WC18 | In this office, supervisors maintain a high standard of ethics and accountability. |  |  |

**Data abstraction form**

| Ser. No. | Key performance indicators | Percentage (%) |
| --- | --- | --- |
| 1 | Contraceptive acceptance rate (CAR) |  |
| 2 | Antenatal care |  |
| 3 | Skilled birth attendance |  |
| 4 | Postnatal care |  |
| 5 | Full immunization services |  |
| 6 | Growth monitoring services |  |
| 7 | Average maternal and child health service  key performance indicator score |  |
