# Supplementary material for: Comparison of maternal and child health service performances following a leadership, management, and governance intervention in Ethiopia: a propensity score matched analysis
Source: BMC Health Serv Res. 2021 Aug 23;21:862. doi: 10.1186/s12913-021-06873-8 (PMC8383359; doi:10.1186/s12913-021-06873-8)
Supplement: Supplementary file 4 — Additional file 4. Propensity matched score analysis, September 2018. [file 12913_2021_6873_MOESM4_ESM.docx]

**Additional file 4: PSM balances property of LMG exposed and non-exposed participants**

| Covariates | Before propensity score matching | | | After propensity score matching | | |
| --- | --- | --- | --- | --- | --- | --- |
|  | Mean score of LMG trainees | Mean score of non-LMG trainees | Std. mean  diff. | Mean score of LMG trainees | Mean score of non-LMG trainees | Std. mean  diff. |
| N | 272 | 272 |  | 227 | 227 |  |
| Propensity score | .545 | .455 | .628 | .513 | .492 | .142 |
| Male | .743 | .739 | .008 | .753 | .740 | .030 |
| Female | .257 | .261 | -.008 | .247 | .260 | -.030 |
| Married | .632 | .665 | -.068 | .621 | .648 | -.055 |
| Separated | .018 | .007 | .082 | .013 | .009 | .033 |
| BSc public health | .202 | .250 | -.119 | .198 | .203 | -.011 |
| Diploma | .449 | .445 | .007 | .476 | .480 | -.009 |
| MSc in health service management | .048 | .044 | .017 | .040 | .040 | .000 |
| Lab and pharmacy | .051 | .051 | .000 | .062 | .053 | .040 |
| Midwife | .110 | .085 | .082 | .110 | .101 | .028 |
| Age 31 – 40 years | .169 | .272 | -.274 | .194 | .247 | -.141 |
| Age 41 – 50 years | .051 | .059 | -.033 | .031 | .035 | -.020 |
| Age 51+ years | .011 | .040 | -.281 | .000 | .000 | .000 |
| Service 6 – 21 years | .382 | .507 | -.257 | .423 | .480 | -.118 |
| Service 22+ years | .037 | .000 | .195 | .000 | .000 | .000 |
| Population | 33948.721 | 36138.434 | -.089 | 33967.432 | 35648.022 | -.068 |
| Distance in KM | 14.551 | 15.182 | -.048 | 14.313 | 14.907 | -.045 |
| Access to road (Yes/ No) | .949 | .971 | -.100 | .978 | .974 | .020 |
| Salary 3,501 – 8,540 ETB | .577 | .603 | -.052 | .599 | .595 | .009 |
| Salary 8,541+ ETB | .088 | .114 | -.091 | .066 | .088 | -.078 |
| Health center | .882 | .846 | .114 | .885 | .863 | .068 |
| Woreda | .015 | .136 | -1.006 | .018 | .115 | -.804 |
