# Supplementary material for: Comparison of maternal and child health service performances following a leadership, management, and governance intervention in Ethiopia: a propensity score matched analysis
Source: BMC Health Serv Res. 2021 Aug 23;21:862. doi: 10.1186/s12913-021-06873-8 (PMC8383359; doi:10.1186/s12913-021-06873-8)
Supplement: Supplementary file 6 — Additional file 6. Levene’s test of equality of variances, September 2018. [file 12913_2021_6873_MOESM6_ESM.docx]

**Additional file 6**: **Levene’s test of equality of variances**

|  | Levene's test for equality of variances | |
| --- | --- | --- |
|  | F | Sig. |
|  |  |  |
| Contraceptive acceptance rate | .095 | .758 |
| Antenatal care | 2.373 | .124 |
| Skilled delivery | 1.673 | .197 |
| Postnatal care | .258 | .612 |
| Full immunization | .362 | .548 |
| Growth monitoring | .245 | .621 |
| Overall performance | .001 | .985 |
| Strengthening management system | 23.319 | .001 |
| Enhanced work climate | 40.707 | .001 |
| Capacity to respond to new challenges (responsiveness) | 14.785 | .001 |
| Overall health systems strengthening | 22.043 | .001 |
